# Assessment of Drug-related Problems among Patients of Chronic liver Disease in a Tertiary Care Hospital

**DOI:** 10.4314/ejhs.v34i6.5

**Published:** 2024-11

**Authors:** M J S Twinkle, Rahifa Ziyad Karjal, A Anushree, Adhiti Kellarai, Bipin Shaji, Shraddha Shetty, P Ramkumar, Juno Jerold Joel

**Affiliations:** 1 Nitte (Deemed to be University), NGSM Institute of Pharmaceutical Sciences (NGSMIPS), Department of Pharmacy Practice, Deralakatte, Mangaluru, India; 2 Nitte (Deemed to be University), KS Hegde Medical Academy (KSHEMA), Department of General Medicine, Deralakatte, Mangaluru, India; 3 Nitte (Deemed to be University), NGSM Institute of Pharmaceutical Sciences (NGSMIPS), Department of Pharmaceutics, Deralakatte, Mangaluru, India; 4 Nitte (Deemed to be University), KS Hegde Medical Academy (KSHEMA), Department of Biostatistics, Deralakatte, Mangaluru, India

**Keywords:** Chronic Liver Disease, Child-Pugh score, Multimorbidity, Polypharmacy

## Abstract

**Background:**

Chronic Liver Disease (CLD) is a long-term condition marked by a gradual decline in liver function. Patients with CLD often experience multimorbidity and polypharmacy, which can adversely affect their health outcomes. The objective of the current study is to identify and resolve the drug-related problems associated with chronic liver disease.

**Methods:**

This prospective observational study involved 150 patients with CLD over a six-month period. Eligible participants included individuals over 18 years old, diagnosed with CLD based on the Child-Pugh score, and currently receiving treatment. Drug-related problems (DRPs) were identified using the Pharmaceutical Care Network Europe (PCNE) classification version 9.1. Data analysis was conducted using Chi-square and Fisher's exact tests with SPSS software version 29.

**Results:**

A total of 212 DRPs were identified and resolved. The most frequent type of DRP was related to treatment efficacy, with 96 instances (45.29%). Within this category, the subcategory ‘effect of drug treatment not optimal’ was the most common, accounting for 45 patients (21.23%). Drug interactions were identified as the leading cause of DRPs, comprising 65 cases (30.66%). Most issues were addressed at the prescriber level, with 48.11% of interventions accepted by physicians.

**Conclusion:**

This study provides valuable insights into identifying and managing DRPs that can negatively impact treatment outcomes in CLD patients. The findings can assist healthcare professionals in prioritizing strategies to enhance clinical results.

## Introduction

Chronic liver disease (CLD) is characterized by a persistent decline in liver function, impacting essential processes such as the production of clotting factors, detoxification of metabolic wastes, and bile excretion. Advanced stages of CLD, such as cirrhosis and fibrosis, result from prolonged inflammation and liver damage, often leading to regeneration challenges. Common symptoms include fatigue, anorexia, and weight loss, though manifestations vary based on disease severity (1,2).

Globally, approximately 1.5 billion individuals are affected by CLD across various stages. Non-alcoholic fatty liver disease (NAFLD) accounts for the majority of cases (59%), followed by hepatitis B (29%), hepatitis C (9%), and alcoholic liver disease (2%) (3). In India, alcohol consumption significantly contributes to liver disease-related mortality. The World Health Organization (WHO) reported age-standardized death rates from CLD of 45.8 per 100,000 for adult men and 14.7 for adult women in 2016 (4). The severity of CLD is evaluated using the Child-Pugh score, which incorporates clinical indicators such as ascites, hepatic encephalopathy, prothrombin time (or INR), serum bilirubin, and albumin levels (5).

CLD treatment involves various medications, including diuretics (e.g., spironolactone and furosemide), nonselective beta-adrenergic blockers for primary prophylaxis, octreotide for variceal bleeding, and long-term antibiotic prophylaxis for spontaneous bacterial peritonitis (SBP) (6). While these medications play a crucial role in managing the disease, they can also lead to drug-related problems (DRPs).

DRPs refer to events or circumstances associated with drug therapy that hinder desired health outcomes. This study employs the PCNE Classification v9.1 to assess the impact of DRPs on treatment efficacy (7). For hospitalized patients, DRPs pose significant safety risks, potentially diminishing quality of life, prolonging hospital stays, increasing costs, and elevating morbidity and mortality rates (8). ADRs, drug-drug interactions, and drug-disease interactions are among the various forms of DRPs. The prevalence of multimorbidity and polypharmacy in CLD underscores the importance of identifying and addressing DRPs to mitigate their adverse health effects (9,10). This study aims to identify and resolve drug-related problems associated with chronic liver disease.

## Materials and Methods

**Study site, design, and duration**: This prospective observational study was conducted at the Department of General Medicine, Justice KS Hegde Charitable Hospital, Mangalore, Karnataka, India, over six months (October 2022 to April 2023). Participants included patients over 18 years old diagnosed with chronic liver disease and undergoing treatment. Exclusions were made for patients in the Intensive Care Unit, obstetric patients, and individuals with psychiatric illnesses. Informed consent was obtained from all participants. Diagnosis of CLD was based on clinical signs of chronic liver disease (e.g., clubbing, palmar erythema, distended abdominal veins, encephalopathy, splenomegaly, ascites), impaired liver function tests (elevated liver enzymes, elevated INR, low serum albumin), and ultrasound evaluations (size, shape, and texture of the liver, presence of cholestasis, spleen size, or ascites).

**Sample size**: Based on the expected proportion of 0.487, a precision of 0.08, at 95% confidence interval, the required sample size was calculated as 150.

**Study procedure**: The research team conducted daily visits to the general medicine wards, reviewing the case files of consenting patients and conducting interviews to identify potential DRPs. Data collected included demographic information, clinical history, biochemical markers, and outcomes. Identified DRPs were categorized according to the PCNE DRP classification version 9.1. These issues were communicated to the treating physician, and the acceptance of interventions was recorded. A structured data collection form was utilized, and data were documented using Microsoft Excel 2019, followed by statistical analysis using Chi-square and Fisher's exact tests in SPSS software version 29.

**Statistical analysis**: Statistical analysis was conducted using the chi-square test in SPSS software version 29. Descriptive statistics such as mean and standard deviation were employed to summarize the data. A p-value of less than 0.05 was considered statistically significant.

**Ethical considerations**: Ethical approval was obtained from the Institutional Ethics Committee, Nitte (Deemed to be University) (Ref. No: NGSMIPS/IEC/017/2022). The study is registered with the Clinical Trial Registry of India (Ref. No.: CTRI/2022/11/047165).

## Results

**Sociodemographics of the study population**: The study, conducted between October 2022 and April 2023, enrolled 150 patients who met the inclusion criteria (Table 1). The mean age of participants was 52.31 ± 11.75 years, with a higher prevalence of the disease observed in males (90%) compared to females.

**Table 1:** Sociodemographic characteristics of patients enrolled in the study

Variables	Categories	n (%)
**Sex**	Male	135 (90)
	Female	15 (10)
**Age (in years)**	< 30 years	5 (3.33)
	31-40 years	19 (12.67)
	41-50 years	35 (23.33)
	51-60 years	53 (35.33)
	61-70 years	31 (20.67)
	71-80 years	7 (4.67)
**Education**	Illiterate	4 (2.67)
	Primary School	17(11.33)
	Middle School	27 (18.00)
	High School	48 (32.00)
	Intermediate	20 (13.30)
	Graduate	34 (22.67)
**Domiciliary status**	Rural	114(76.00)
	Urban	36 (24.00)
**Social Habits**	None	58 (38.67)
	Alcohol	73 (48.67)
	Alcohol and smoking	14(9.33)
	Alcohol and tobacco	4 (2.67)
	Alcohol, smoking, and tobacco	1 (0.67)
**Comorbidities**	None	65 (43.33)
	Hypertension	25 (16.67)
	Diabetes mellitus	35 (23.33)
	Hypertension and Diabetes mellitus	22 (14.67)
	Thyroid disease	2(1.33)
	Asthma	1 (0.67)
**Number of Drugs**	<5	25 (16.66)
	>5	125 (83.33)
**Length of hospital stay**	1-5	70 (46.67)
	6-10	53 (35.33)
	11-15	16 (10.67)
	16-20	11 (7.33)

Variables were analyzed in relation to drug-related problems (DRPs), and the Child-Pugh score was utilized to assess disease severity. Most participants presented with moderate disease severity. A significant association was found between DRP status and factors such as the number of medications, length of hospital stay, and comorbidities, as determined by the chi-square test (Table 2).

**Table 2:** Factors associated with drug-related problems

Variables	Patients without DRP	Patients with DRP	Significance
**Comorbidities**			
Without	27 (60.0)	37(35.2)	P-value = 0.005
With	18(40.0)	68(64.8)	
**No. of drugs**			
0-10	42(93.3)	45(42.9)	
11-20	3(6.7)	57(54.3)	P-value<0.001
>20	0(0.0)	3(2.9)	
**Length of stay**			
0	17(37.8)	0(0.0)	
0-5	17(37.8)	36(34.3)	
6-10	11(24.4)	42(40.0)	P-value <0.001
11-15	0(0.0)	16(15.2)	
16-20	0(0.0)	11(10.5)	

**Classification of study population based on severity**: The severity of the disease was classified using the Child-Pugh Score. Among the 150 patients enrolled in the study, 64 (42.67%) were categorized as Child-Pugh class B, followed by 60 (40.00%) in class C. The least represented group was Child-Pugh class A, with 26 patients (17.33%).

**Management of the disease**: The therapeutic management of liver disease and its associated comorbidities—including hypertension, diabetes mellitus, asthma, and hyperthyroidism—utilized medications such as propranolol, metformin, budesonide (administered via nebulizer), and Carbimazole. The most frequently prescribed antibiotic was rifaximin (Table 3).

**Table 3:** Medication usage pattern in the study population

Category of medication	Type of medication	Frequency	Percent
**Antihypertensive drugs**	Tab. Spironolactone + Furosemide	98	65.3
	Tab. Spironolactone	40	26.6
	Tab.Propranolol	135	90
	Tab.Telmisartan	40	26.6
	Tab.Losartan	35	23.3
	Tab. Furosemide	12	8
	Tab.Cilnidipine	15	10
	Tab. Clonidine	22	14.6
	Tab. Torsemide+Spironolactone	26	17.3
	Tab. Torsemide	12	8
	Tab. Carvedilol	16	10.6
	Tab.Metoprolol	18	12
	Tab. Midodrin	119	79.3
**Antidiabetic drugs**	Tab.Sitagliptin +Metformin	6	4
	Tab.Metformin	15	10
	Tab.Sitagliptin	8	5.3
	Tab.Gliclazide	7	4.6
	Tab.Teneligliptin	6	4
	Insulin. Human Mixtard	10	6.6
	Tab. Vildagliptin	5	3.3
**Antibiotics**	Tab. Rifagut	143	95.3
	Inj. Meropenem	102	68
	Inj. Ceftriaxone	97	64.6
	Inj. Piptaz	98	0.6
	Inj. Augmentin	79	52.6
	Inj. Metronidazole	70	46.6
	Tab. Clindamycin	60	40
	Tab. Cefixime	52	34.6
**Electrolyte supplement**	Syp. Potassium Chloride	127	84.6
**Hepatoprotectives**	Tab. Ursodeoxycholic Acid	137	91.3
	Tab. Ademethionine	131	87.3
	Tab. Silymarin	55	36.6
**NSAIDS**	Tab. Diclofenac	43	28.6
**Analgesics and Antipyretic**	Tab. Acetaminophen	76	50.6
**Hepatic encephalopathy**	Syp. Lactulose	135	90
	Tab. L Ornithine L Aspartate	73	48.6
	Tab. Pancreatin	62	41.3
**Ulcer protectants**	Tab. Esomeprazole and Domperidone	67	44.6
	Tab. Ondansetron	54	36
	Tab. Pantoprazole	97	64.6
	Syp. Sucralfate + Oxetacaine	134	89.3
**Plasma Volume Expander**	Inj. Albumin	73	48.6

**Classification of DRPs according to PCNE 9.1**: Using the Pharmaceutical Care Network Europe (PCNE) classification version 9.1, the identified drug-related problems (DRPs) were categorized into various types and causes. A total of 212 DRPs were identified. The predominant type was “treatment safety” (P2), comprising 94 cases (44.34%), followed closely by “treatment effectiveness,” which included 96 instances (45.29%). Within the “treatment effectiveness” category, the most frequent subcategory was “effect of drug treatment not optimal” (P1.2), with 45 cases (21.23%).

Among the various causes of DRPs identified in the study, “drug selection” (C1) emerged as the most common, with “inappropriate combination of drugs” (C1.3) being the leading subcategory, accounting for 65 cases (30.66%). The second most common cause was “no or incomplete drug treatment despite existing indications,” with 24 cases (11.32%).

Interventions for the 212 DRPs were primarily conducted at the prescriber level, with 139 interventions (65.56%) being prescriber-informed only. Most interventions for the identified DRPs were accepted but not implemented (A1.3), totaling 110 cases (51.88%). Overall, 48.11% of interventions were accepted by physicians, resulting in the resolution of the DRPs (Table 4).

**Table 4:** DRPs as per PCNE classification version 9.1

Code	Detailed classification	n (%)
**P1**	**Treatment effectiveness**	**96 (45.29)**
**P1.1**	No effect of drug treatment despite correct use	23 (10.85)
**P1.2**	Effect of drug treatment not optimal	45 (21.23)
**P1.3**	Untreated symptoms or indication	28(13.21)
**P2**	**Treatment safety**	**94 (44.34)**
**P2.1**	Adverse drug event (possibly) occurring	94 (44.34)
**P3**	**Other**	**22(10.37)**
**P3.1**	Unnecessary drug-treatment	14 (6.60)
**P3.2**	Unclear problem/complaint. Further clarification necessary	8 (3.77)
**C1**	**Drug selection**	**127(59.9)**
**C1.2**	No indication of drug	8 (3.77)
**C1.3**	Inappropriate combination of drugs, or drugs and herbal medications, or drugs and dietary supplements	65 (30.66)
**C1.4**	Inappropriate duplication of therapeutic group or active ingredient	11 (5.19)
**C1.5**	No or incomplete drug treatment in spite of existing indication	24 (11.32)
**C1.6**	Too many different drugs/active ingredients prescribed for indication	19 (8.96)
**C2**	**Drug form**	**2(0.94)**
**C2.1**	Inappropriate drug form/formulation	2 (0.94)
**C3**	**Dose selection**	**62(29.24)**
**C3.1**	Drug dose too low	8 (3.77)
**C3.2**	Drug dose of a single active ingredient too high	8 (3.77)
**C3.3**	Dosage regimen not frequent enough	13 (6.13)
**C3.4**	Dosage regimen too frequent	21 (9.91)
**C3.5**	Dose timing instructions wrong, unclear or missing	12 (5.66)
**C4**	**Treatment duration**	**21(9.9)**
**C4.1**	Duration of treatment too short	18(8.49)
**C4.2**	Duration of treatment too long	3(1.42)
**A1**	**Intervention accepted**	
**A1.1**	Intervention accepted and fully implemented	102 (48.11)
**A1.3**	Intervention accepted but not implemented	110(51.88)

In patients with hepatic impairment (Child-Pugh class C), the dosage of losartan tablets is adjusted to 25 mg once daily, down from the standard 40 mg. To prevent hepatic encephalopathy, rifaximin is prescribed at a dosage of 550 mg twice daily or 400 mg three times daily. No dosage adjustment is necessary for rifaximin; however, monitoring is recommended in cases of severe impairment (Child-Pugh class C).

The standard dose of metronidazole injection is 500 mg every 8 hours, but this is reduced to 500 mg every 12 hours for children with Child-Pugh class C. Acetaminophen, an antipyretic and analgesic medication, is typically administered at a dose of 500-1000 mg every 4-6 hours. In patients with hepatic impairment, the dosage should be minimized and not exceed 2 grams per day (Table 5).

**Table 5:** Potential medications that need hepatic dose adjustment

Drug name	Formulation	Indication	Standard dose	Standard Hepatic adjusted dose	Dose administered
**Losartan**	Tablet	For the treatment of hypertension	40 mg once daily	25 mg once daily	25 mg once daily (monitored in patients with ascites)
**Rifaximin**	Tablet	To prevent hepatic encephalopathy	550 mg twice daily or 400 mg three times daily	No dose adjustment needed	Monitoring is required in severe impairment (Child-Pugh class C)
**Metronidazole**	Injection	Antibiotic therapy	500 mg every 8 hours	For child-pugh class c, the daily dose is reduced to 50%	500 mg every 12 hours
**Acetaminophen**	Tablet	Antipyretic and Analgesic	500 mg to 1000 mg every 4 to 6 hours or as required	650 mg to 2gm, reduce the dose to the maximum, not more than 2gm/day)	650 mg whenever required (reduced the dose to the maximum not exceeded 2gm/day)

Major drug interactions: Significant drug interactions were found between diuretics (spironolactone) and electrolyte supplements (potassium chloride), diuretics (Furosemide) and ulcer protectants (Sucralfate), diuretics (furosemide) and Non-Steroidal Antiinflammatory Drugs (NSAIDs) (Diclofenac). The drug interactions were identified using the UpToDate software (Table 6).

**Table 6:** Major drug-drug interactions identified

Drugs involved	Severity	Risk Rating	Outcomes
**Spironolactone + Potassium chloride**	Major	X	Hyperkalemia
**Furosemide+ Sucralfate**	Major	D	This combination decreases the concentration of furosemide
**Furosemide + Diclofenac**	Major	D	This combination diminishes the effect of furosemide
**Flucanozole+ Domperidone**	Major	X	Enhances the QT prolongation
**Tramadol+ Lorazepam**	Major	D	Enhances CNS depression
**Gliclazide+ Teneligliptin**	Major	D	Hypoglycemia

X: To be avoided since the risks usually outweigh the benefits; D: Consider therapy modification.

**Adverse drug reactions**: During the study period, a total of 10 adverse drug reactions (ADRs) were reported, and their causality was assessed using the Naranjo Adverse Drug Reaction Probability Scale. The adverse drug reactions (ADRs) identified during the study were as follows:

Propranolol: Constipation (Probable), and Tingling sensation (Probable) observed in one patient each. Ursodeoxycholic acid: Constipation (Probable), observed in one patient. Pantoprazole: Vomiting (Probable) and Thrombocytopenia (Probable), each observed in one patient. Spironolactone: Hyperkalemia (Possible), observed in one patient. Vildagliptin: Skin rash (Probable), observed in one patient and Furosemide: Hyponatremia (Possible), observed in one patient. The most commonly identified ADRs included constipation, hyponatremia, and hyperkalemia.

## Discussion

The present study employed the PCNE Classification v9.1 to assess how chronic liver disease impacts treatment effectiveness in 150 patients. The aim was to analyze various types of drug-related problems (DRPs) in these patients, identify their underlying causes, and evaluate the efficacy of treatments in resolving these issues.

The findings revealed a predominance of males over females, consistent with the observations of Sagnelli et al., which may be attributed to higher rates of alcohol consumption among men. The mean age of the patient population was 52.31 ± 11.75 years, aligning with the findings of Sajja et al. Ageing has been identified as a significant risk factor, as the majority of DRPs in our study were associated with individuals over 50 years of age. Regarding educational status, most participants had completed high school, which contrasts with Ghamdi et al., where the majority were college graduates. A lower educational level has been linked to a higher burden of alcohol-related diseases.

Type II diabetes mellitus (DM) was the most common comorbidity in our study population, similar to findings by Afaf Mohamed et al., who reported that 76.3% of chronic liver disease patients have DM due to impaired glucose metabolism. Most patients in our study were alcoholics. Globally, excessive alcohol consumption is a leading cause of liver cirrhosis, underscoring its significance as a major etiological factor. While the underlying causes of cirrhosis are generally consistent worldwide, their relative contributions vary by region, a finding echoed in a study by Vaz et al., which found that 50.5% of its patients were alcoholics.

A significant portion of our study population exhibited moderately impaired liver function, as indicated by Child-Pugh class B. This is likely because patients often do not seek treatment until their condition has significantly progressed, as early stages frequently lack noticeable symptoms. Similar results were reported by Jonathan et al., who found that 49.5% of patients fell into the Child-Pugh B category.

Using the PCNE Classification of DRPs v9.1, we identified a total of 212 DRPs among 105 patients. The most common issue was treatment effectiveness, particularly the subcategory “effect of drug treatment not optimal” (P1.2), which accounted for 21.23% of the DRPs. In contrast, Movva et al. reported different results using PCNE Classification v6.2, with the most common issue being the wrong effect of drug treatment (24.7% of DRPs), followed by the suboptimal effect of drug therapy (20.4%). Similarly, Saldanha et al. found that common DRPs included issues related to drug usage processes and treatment ineffectiveness.

Among the identified DRPs, the most frequent cause was drug selection (C1), with the subcategory “inappropriate combination of drugs” (C1.3) accounting for 30.66%. This prevalence can be attributed to polypharmacy, which increases the risk of drug interactions and adverse effects. This finding aligns with Movva et al., who noted that medication selection issues were a significant source of problems (22.17%).

Most interventions for the 212 DRPs were conducted at the prescriber level, with 65.56% being prescriber-informed. Similar results were observed by Kumar et al., where 63% of interventions occurred at this level. Movva et al. noted that most interventions suggested adding medications, followed by recommendations for discontinuation.

In our study, most interventions were accepted but not implemented (A1.3). Although the suggestions had merit, concerns regarding their relevance to the patient's primary condition or the need for careful observation before making therapeutic changes may have led physicians to delay implementation. This is consistent with findings from Movva et al., where 68.26% of accepted interventions did not result in action. Conversely, Ceylan et al. found that most interventions were fully accepted and implemented.

The Chi-square test of independence in our study revealed statistically significant associations between DRP status and factors such as length of hospital stay. This finding was corroborated by Wincent et al., who also found a significant relationship between length of stay and DRP status. However, Movva et al. reported no such association. Our study also found significant correlations between the number of medications and comorbidities with DRP status, consistent with findings from Noe Garin et al., which identified polypharmacy as a significant risk factor for DRPs. The increased medication burden during hospitalization can exacerbate polypharmacy, leading to potential drug-drug interactions and necessitating vigilant monitoring.

The strengths of this study include comprehensive follow-up until patient discharge, as well as the identification and resolution of drug interactions and adverse drug reactions in consultation with physicians. However, limitations include the study's single-center design, small sample size, short duration, and the lack of cost analysis for therapy.

In conclusion, the study identified 212 drug-related problems (DRPs) among patients with chronic liver disease (CLD) and decompensated chronic liver disease (DCLD). The findings highlight critical aspects of treatment effectiveness and safety, providing valuable insights for improving therapeutic outcomes. Implementing clinical pharmacy services in routine practice is essential for optimizing therapy.

## Figures and Tables

**Figure 1:**
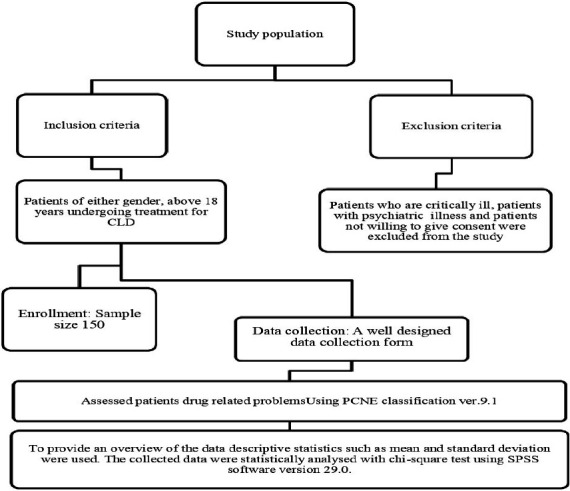
Flow chart of the methodology followed in the study

## References

[1] Sharma A, Nagalli S (2023). Chronic Liver Disease. StatPearls [Internet].

[2] Zawdie B, Tadesse S, Wolide AD, Nigatu TA, Bobasa EM (2018). Non-alcoholic fatty liver disease and associated factors among type 2 diabetic patients in Southwest Ethiopia. Ethiopian Journal of Health Sciences.

[3] Cheemerla S, Balakrishnan M (2021). Global Epidemiology of Chronic Liver Disease. Clin Liver Dis.

[4] Sarin SK, Kumar M, Eslam M, George J (2020). Liver diseases in the Asia-Pacific region: a Lancet Gastroenterology & Hepatology Commission. Lancet Gastroenterol Hepatol.

[5] Tsoris A, Marlar CA (2023). Use of the Child-Pugh Score in Liver Disease. StatPearls.

[6] Dipiro J T, Talbert R L, Yee G C (2017). Pharmacotherapy a pathophysiologic approach.

[7] Classification for Drug related problems [ebook] Pharmaceutical Care Network Europe Association.

[8] Bhagavathula SA, Meknonnen BG, Birarra KM, Tekle TM (2017). Assessment of Drug Related Problems and its Associated Factors among Medical Ward Patients in University of Gondar Teaching Hospital, Northwest Ethiopia: A Prospective Cross-Sectional Study. J Basic Clin Pharma.

[9] Hayward Kelly L, Weersink Rianne A (2020). Improving Medication-Related Outcomes in Chronic Liver Disease. Hepatology Communications.

[10] Aghili M, Neelathahalli Kasturirangan M (2019). Identifying characteristics of drug-related problems in critically ill patients with decompensated liver cirrhosis. Eur J Gastroenterol Hepatol.

[11] Sagnelli E, Stroffolini T, Sagnelli C (2018). Gender differences in chronic liver diseases in two cohorts of 2001 and 2014 in Italy. Infection.

[12] Sajja KC, Mohan DP, Rockey DC (2014). Age and ethnicity in cirrhosis. J Investig Med.

[13] Kim IH, Kisseleva T, Brenner DA (2015). Aging and liver disease. Curr Opin Gastroenterol.

[14] Al Ghamdi SS, Shah H (2018). An Educational Needs Assessment for Patients with Liver Disease. J Can Assoc Gastroenterol.

[15] Mohamed AM, Isa HM (2020). Health related quality of life in patients with chronic diseases. International Journal of Medicine and Public Health.

[16] Duah A, Agyei-Nkansah A, Osei-Poku F, Duah F, Addo BP (2021). Sociodemographic characteristics, complications requiring hospital admission and causes of in-hospital death in patients with liver cirrhosis admitted at a district hospital in Ghana. PLoS One.

[17] Vaz J, Eriksson B, Strömberg U (2020). Incidence, aetiology and related comorbidities of cirrhosis: a Swedish population-based cohort study. BMC Gastroenterol.

[18] Stine IG, Stukenborg GJ, Wang I, Adkins A, Niccum B, Zimmet A, Argo CK (2020). Liver transplant candidates have impaired quality of life across health domains as assessed by computerized testing. Annals of hepatology.

[19] Movva R, Jampani A, Nathani I, Pinnamaneni SH, Challa SR (2015). A prospective study of incidence of medication-related problems in general medicine ward of a tertiary care hospital. J Adv Pharm Technol Res.

[20] Saldanha V, Randall Martins R, Lima SIVC, Batista De Araujo I (2020). Incidence, types and acceptability of pharmaceutical interventions about drug related problems in a general hospital: An open prospective cohort. BMJ Open.

[21] Farooq I, Sana MM, Chetana PM, Almuqbil M, Prabhakar Bhat N, Sultana R, Khaiser U, Mohammed Basheeruddin Asdaq S, Almalki MEM, Mohammed Sawadi Khormi A, Ahmad Albraiki S, Almadani ME (2023). Polypharmacy in chronic liver disease patients: Implications for disease severity, drug-drug interaction, and quality of life. Saudi Pharm J.

[22] Kumar MV, Pasha MM, Varshith G, Pravalika B, Shameela M (2022). Drug Related Problems Identified by Clinical Pharmacist in a Tertiary Care Hospital in South India: An Observational Study. Indian Journal of Pharmacy Practice.

[23] Ceylan C, Sancar M, Beceren A, Demir A, Kuş C, ve Omurtağ G Z (2022). Evaluation of clinical pharmacist interventions on drug-related problems in the gastroenterology ward. Journal of Research in Pharmacy.

[24] Wincent M M, Potrilingam D, V. A, Jacob S C, G. A (2017). “assessment of drug related problems in patients with chronic diseases in the general medicine units of a tertiary care hospital”. International Pharmacy Journal.

[25] Garin N, Sole N, Lucas B, Matas L, Moras D, Rodrigo-Troyano A, Gras-Martin L, Fonts N (2021). Drug related problems in clinical practice: a cross-sectional study on their prevalence, risk factors and associated pharmaceutical interventions. Scientific reports.

[26] Kefale B, Degu A, Tegegne GT (2020). Medication-related problems and adverse drug reactions in Ethiopia: A systematic review. Pharmacology research & perspectives.

